# Global View of Candidate Therapeutic Target Genes in Hormone-Responsive Breast Cancer

**DOI:** 10.3390/ijms21114068

**Published:** 2020-06-06

**Authors:** Annamaria Salvati, Valerio Gigantino, Giovanni Nassa, Valeria Mirici Cappa, Giovanna Maria Ventola, Daniela Georgia Cristina Cracas, Raffaella Mastrocinque, Francesca Rizzo, Roberta Tarallo, Alessandro Weisz, Giorgio Giurato

**Affiliations:** 1 Laboratory of Molecular Medicine and Genomics, Department of Medicine, Surgery and Dentistry ‘Scuola Medica Salernitana’, University of Salerno, 84081 Baronissi (SA), Italy; asalvati@unisa.it (A.S.); vgigantino@unisa.it (V.G.); gnassa@unisa.it (G.N.); valeria.mirici@gmail.com (V.M.C.); frizzo@unisa.it (F.R.); rtarallo@unisa.it (R.T.); 2 Genomix4Life, 84081 Baronissi (SA), Italy; giovannaventola3es9@gmail.com (G.M.V.); danielacracas@gmail.com (D.G.C.C.); rmastrocinque@unisa.it (R.M.); 3 CRGS—Genome Research Center for Health, University of Salerno Campus of Medicine, 84081 Baronissi (SA), Italy

**Keywords:** estrogen receptor α, breast cancer, estrogen signaling, endocrine therapy

## Abstract

Breast cancer (BC) is a heterogeneous disease characterized by different biopathological features, differential response to therapy and substantial variability in long-term-survival. BC heterogeneity recapitulates genetic and epigenetic alterations affecting transformed cell behavior. The estrogen receptor alpha positive (ERα+) is the most common BC subtype, generally associated with a better prognosis and improved long-term survival, when compared to ERα-tumors. This is mainly due to the efficacy of endocrine therapy, that interfering with estrogen biosynthesis and actions blocks ER-mediated cell proliferation and tumor spread. Acquired resistance to endocrine therapy, however, represents a great challenge in the clinical management of ERα+ BC, causing tumor growth and recurrence irrespective of estrogen blockade. Improving overall survival in such cases requires new and effective anticancer drugs, allowing adjuvant treatments able to overcome resistance to first-line endocrine therapy. To date, several studies focus on the application of loss-of-function genome-wide screenings to identify key (hub) “fitness” genes essential for BC progression and representing candidate drug targets to overcome lack of response, or acquired resistance, to current therapies. Here, we review the biological significance of essential genes and relative functional pathways affected in ERα+ BC, most of which are strictly interconnected with each other and represent potential effective targets for novel molecular therapies.

## 1. Introduction

Molecular heterogeneity and complexity make breast cancer (BC) one of the most aggressive tumors with a high mortality rate worldwide. BC categories obtained from gene expression profiles allowed a recognized classification of this pathology in four major subtypes comprising the estrogen receptor-positive (luminal A and luminal B), HER2-positive, normal-like and triple-negative breast cancers (TNBC) [1]. The estrogen receptor alpha (ERα) is the key factor in modulating estrogen signaling by its oncogenic effect in over 70% of luminal breast carcinoma, and its presence justifies, in this biological context, the endocrine therapy approach. In clinical practice, in fact, this therapeutic regimen has led to increasing patients’ overall survival, representing nowadays the most effective treatment for ERα positive (ERα+) BC. The development of endocrine therapies is pivoted on blocking estrogen-dependent growth of luminal BC cells determining anti-proliferative and apoptotic mechanisms through ERα functional inhibition [2]. Endocrine therapy strategies are mainly based on the use of Selective Estrogen Receptor Modulators (SERMs), such as tamoxifen or raloxifene, and Selective Estrogen Receptor Downregulators (SERDs), such as Fulvestrant (or ICI 182780) that act, respectively, as partial and pure antagonists of ERα. On the other hand, Aromatase Inhibitors (AIs) are the first-line treatment for postmenopausal women acting upstream of the production of estrogens, inducing the block of their peripheral conversion from androgens [3]. Unfortunately, the development of endocrine therapy resistance occurs in over 50% of patients sustained by de novo or acquired resistance, the latter, emerging after first-line treatments or in relapse cases. This condition is often due to combination of genetic or epigenetic alterations, such as dysregulation of growth factor pathways crosstalking with ERα signaling, alterations in chromatin remodeling processes, loss of ERα expression itself, ERα polymorphic variations and modifications in coregulators recruitment to ERα complexes [4]. Nowadays, the advent of new editing engineering tools, associated with the development of sequencing technologies, has led to the large-scale use of genome-scale loss-of-function screenings bringing several benefits in cancer dependency genes identification and thus potential drug targets discovery. Large-scale RNA interference (RNAi) screenings and Clustered Regularly Interspaced Short Palindromic Repeats (CRISPR)-Cas9-based single guide RNA are the two main techniques mainly used to characterize gene function, disrupting their normal expression and analyzing the consequent effects on phenotype [5]. In particular, the genome-wide CRISPR-Cas9 approach offers a wider knowledge of multiple active pathways and complex gene networks within cancer cells, giving the opportunity to characterize the genetic neoplastic landscape and highlight the functions of thousands of genes in the context of their essentiality for cell survival and proliferation. These genes, known as essential genes, can be considered potential candidates for the development of new pharmacological molecules or specific immunotherapy strategies able to counteract cancer driver genes activities. Broad [6] and Welcome Sanger [7] Institutes have performed the two largest independent CRISPR-Cas9-based “dropout” screenings. Despite the distinct experimental procedures and computational approaches used to estimate gene dependency levels, both datasets show extensive overlap and robustness [8]. In this review, we carried out a systematic feature characterization of BC cell lines screened within the Achilles [6] and Sanger Score [7] projects, in order to highlight genes identified with this approach associated with the ERα+ BC phenotype. Concordance between essential genes identified in BC cell lines by screening with either GeCKO CRISPR-Cas [9] or lentiviral-based pooled RNAi libraries [10] was also considered. Moreover, further essential gene datasets comparison was performed starting from two additional CRISPR knockout screens carried out specifically in hormone-responsive BC cell lines [11,12], allowing a systematic genetic characterization of hormone-responsive BC cells for identification of gene essentiality.

Selected essential genes in common between Meyers et al. [6] and Behan et al. [7] dropout datasets were used for functional analyses, allowing to highlight molecular signatures involved in deregulated pathways and, when correlated with ERα-interacting molecular partners in the same cell type, in specific functions of ERα+ in BC. Given the crucial role of this receptor in the hormone-dependent tumors and its relevance as a therapeutic target, data reviewed here may uncover also novel putative biomarkers and molecular targets for the development of new therapeutic strategies inactivating intrinsic oncogenic pathways or inhibiting physical ERα-coregulator interaction, to prevent and/or overcome resistance to current therapies.

## 2. Estrogen Signaling and Endocrine Resistance in Breast Cancer

BC, being a heterogeneous pathology, can be classified through the status determination of the following receptors, Estrogen Receptor alpha (ERα), Progesterone Receptor (PR) and epidermal growth factor receptor 2 (HER2/neu), because they represent known targets for BC treatment. Based on these molecular markers, BC can be further classified into four basic subgroups, i.e., (ERα+|PR+) HER2−, (ERα+|PR+) HER2+, (ERα−|PR−) HER2+, (ERα−|PR−) HER2− [13]. The high incidence of hormone-dependent BC and its molecular profile suggests the key role of hormone receptors. In particular, ERα is mainly involved in propagating estrogen signals, essential for BC development, activating pro-proliferative signals both in normal and pathological conditions. ERα is a 595 amino acid protein encoded by the ESR1 gene on the q24-27 of chromosome 6 [14,15]. Besides full-length ERα transcript, several receptor isoforms, such as ERα 46-kDa and ERα-36, can be generated by frame-shift mutations in the ESR1 gene or by alternative splicing mechanisms [16,17]. Both receptor variants can mediate the membrane-initiated estrogen-dependent activation of mitogenic signaling pathways [18].

The transcriptional modulation of E2-dependent genes by ERα can be regulated through both nongenomic pathways involving the crosstalk of the receptor with different signaling pathways and nuclear genomic mechanisms. The latter, also known as the canonical pathway, ensure the direct ERα-DNA association at palindromic Estrogen Response Element (ERE) sequences within or close to regulatory regions [19] or by ERα-protein interactions (tethering mechanism) as AP1 (Activator Protein 1) or SP1 (Specificity Protein 1) [20]. Target genes’ accessibility to ERα is allowed also by chromatin remodeling factors recruitment, such as the Switch-Sucrose Non-fermentable complex (SWI/SNF). This complex, by ATP hydrolysis, determines the change nucleosome structure, promoting accessibility for coregulating proteins which can act both as corepressors (e.g., NcoR, (Nuclear Receptor coRepressor)) and/or coactivators, (e.g., the transcription factor SRC1, Steroid Receptor Coactivator 1) and the p300/CBP) [21]. These molecular complexes play an important role in the recruitment of transcriptional machinery, in the modulation of chromatin structure, and in the regulation of ER target gene expression, such as c-Myc, c-Fos, E2F1, E2F2, TFF1, GREB1 [22,23], that promote cell growth, proliferation and suppression of apoptotic mechanisms [22].

## 3. Mechanisms of Endocrine Resistance in Breast Cancer

The major issue associated with BC treatment and management is the development of endocrine therapy resistance. Endocrine therapy is based on ERα blockade [2] counteracting its functional activities by using Selective Estrogen Receptor Modulators (SERMs) such as tamoxifen, and Selective Estrogen Receptor Downregulators (SERDs) like Fulvestrant, acting as a partial and pure antagonist of ERα actions [3], respectively. Endocrine therapy resistance is attributed to several mechanisms including loss of ERα expression, altered activity of coregulators, and crosstalk between the ERα and growth factor signaling pathways. ERα mutations also play an important role in the development of resistance mechanisms: for instance, they may lead to a conformational change in the ligand-binding domain, interfering with agonists and antagonists interaction and promoting resistance acquisition, as shown for ERαD538G and ERαY537S receptor variants [24,25,26]. These somatic mutations are detected in about 3–10% of samples, with D538G being the most frequent (about 36%), followed by Y537S (about 14%) [27,28] and they are rarely detected in primary treatment-naïve tumors, suggesting either the clonal selection of resistant clones or their later acquisition under the pressure of drug treatment, as a new mechanism of resistance [29]. Since ESR1 mutations are activating mutations, they represent important treatment targets [30]. A preclinical study of a Y537S ER mutation PDX model demonstrates how Bazedoxifene, a third-generation SERM with SERD activity, as a single agent or in combination with palbociclib, reduces tumor growth [31]. Moreover, drug effects can be different among people and depending upon several genetic factors. Alterations in tamoxifen metabolism have been studied as possible causes of different responses to this therapy [32]. Another important role in triggering resistance to endocrine therapies in BC has been played by growth factors and their receptor signaling pathways, being able to activate ERα in absence of ligand [33]., Recently, has been reported that alterations in chromatin remodeling processes promote resistance to endocrine therapy. Among these, mutations in ARID1A, represent the most common alterations of the SWI/SNF chromatin complex in ERα+ BCs. Cellular plasticity is mediated by loss of ARID1A-dependent SWI/SNF complex targeting to genomic sites of the luminal lineage-determining transcription factors, including ERα, forkhead box protein A1 (FOXA1) and GATA-binding factor 3 (GATA3). ARID1A silencing promotes a switch from a luminal to a basal lineage, contributing to the development of endocrine resistance [34]. Therefore, the identification of the mechanisms of drug resistance will be essential to obtain more specific and effective therapies and, in this context, gene essentiality represents a promising approach.

## 4. Dropout Screening Approaches to Dissect Gene Vulnerabilities

Specific silencing of target gene expression has always been a strategy to define the effects of a loss-of-function gene in biological processes. RNAi is a posttranscriptional gene silencing process through which double-stranded RNA (dsRNA) is directed to bind target mRNA in the cytoplasm, leading to its degradation or temporary inactivation, therefore altering the correspondent gene expression without modifying its nucleotide sequence [35,36,37]. RNAi is widely used as a reverse genetic approach to understanding genetic functions and molecular mechanisms in altered pathways or diseases. The off-target is due to a nonspecific or partial sequence complementarity of mRNA target, representing a limitation that has led scientists to investigate alternative methods such as the use of engineered nucleases. Zinc finger nucleases (ZFNs) and transcription activator-like effector nucleases (TALENs) [38,39] are able to produce double-strand breaks in the sequence of interest, allowing activation of cell repair mechanisms, through both nonhomologous end-joining (NHEJ) and homologous recombination (HR). Despite the multiple potential applications, ZFNs and TALENs technologies have some limitations like the complexity in experimental design, time consumption and impossibility to carry out multiple-gene targeting experiments.

The real revolution in the genome editing field is the most recent implementation of Clustered Regularly Interspaced Short Palindromic Repeats (CRISPR)-Cas technology. CRISPR-Cas is an acquired immune system defense of prokaryotic organisms, mainly developed in bacteria and archaea, to target foreign nucleic acids in viruses and plasmids infection. This adaptive mechanism allows immunity response even in subsequent infections by the same invader [40].

The Type II CRISPR-Cas is currently the most popular system used for genome editing in mammalian cells as it requires only the action of single multidomain Cas protein such as Cas9 and Cas12a endonucleases, both able to generally rely on DNA double-strand breaks (DSBs) by host DNA repair enzymes activation. Cas9 is the first well-characterized enzyme associated with the CRISPR II system and it has been described for the first time in Streptococcus pyogenes [41]; Cas9 is essential both as an effector of CRISPR silencing mechanism and for the maturation of the CRISPR RNA (crRNA) [42]. During the immune response, CRISPR locus, inside which viral genome regions are inserted during the first infection, is transcribed in a long RNA molecule (pre-crRNA), containing repeats and spacers. Pre-crRNA maturation in type II systems requires both the stabilization of long transcript by Cas9 protein and transactivation-RNA (tracrRNA) activity [43]. The combination of crRNA and tracrRNA in a single guide RNA (sgRNA) has further simplified the CRISPR-Cas9 system, developing a genome engineering tool based on only two molecules activity [44].

Nowadays, Cas9 is the programmable DNA nuclease widely used as a genome editing tool in eukaryotic cells because it is more flexible and versatile then ZNF and TALEN enzymes since this unique nuclease can target enormous quantity of specific DNA sites through the coexpression of specific sgRNA. A further advantage of the CRISPR-Cas9 system coupled to next-generation sequencing is to easily identify sgRNA with a specific barcode. A pool of sgRNAs is transfected by lentiviral transduction using a low multiplicity of infection ensuring the penetrance by only a single sgRNA type per cell and minimizing the false-positive discovery rate [45]. This determines the knocking out of thousands of individual genes allowing to check the effects on cell behavior and phenotype. The other advantages are given by the versatility of the CRISPR tool because it is possible to apply it in both in vivo and in vitro models and to engineer the Cas enzyme. In CRISPR-mediated repression (CRISPRi), for example, dCas9, characterized by absent catalytic activity, is able to disturb RNA polymerase recruitment or other transcriptional factors after its binding to the target site, determining silence of the gene. On the contrary, in CRISPR-mediated activation (CRISPRa) libraries, fusion enzyme between dCas9 and a repeating peptide array transcription factor enhances the transcriptional activity [46].

Despite the promising advantages and many experimental uses, this technology also shows some drawbacks. For example, in dropout screening, knockout gene efficiency is variable and this depends on several factors, such as sgRNA design, DNA target site, PAM sequence or frequency of double-strand repair pathways activity. The off-target cutting can be the consequence of sequence similarity between sgRNA and the target site. In order to increase the targeting specificity of the CRISPR-Cas9 system, several approaches have been developed to overcome off-target effects. To this aim, bioinformatics tools offer a guide to creating an optimal sgRNA including CRISPR design, E-CRISPR, and CROP-IT [47]. Moreover, the detection of off-target sites can be performed by whole-genome sequencing (WGS) but high cost and time consuming made necessary the development of genome-wide tools able to enrich the sites that undergo DSB, such as Guide-seq, DIAGENOME-seq, DISCOVER-seq or SITE-Seq [47,48,49]. In addition, other algorithms for on- and off-target activity predictions have been implemented, allowing further optimization of genome-wide libraries [50].

Despite its limitations, CRISPR-Cas9 applications as genome editing and genome screening tools enhance the ability to perform high-throughput analyses of gene functions, becoming nowadays the gold standard for driver-disease gene identification, therapy development and drug target gene screening. The latter can be also associated with chemogenomic screening sets containing small molecules that have well-annotated drugs and that are suitable for phenotypic screenings.

High-throughput screening is an approach for accelerating drug discovery by processing large compound libraries at a rate that may exceed a few thousand compounds per day or per week [51,52]. The integration of CRISPR-Cas9 loss-of-function screenings coupled to chemogenomic ones addresses the identification of new potential therapeutic targets in cancers [53]. This reveals new uses for existing drugs, yet utilized in clinical practice and to classify the toxic mechanisms of new compounds.

Regarding the several ways developed to approach CRISPR-Cas9 data, different algorithms have been implemented. They try to address those issues that still influence them, such as the variability in guide RNA efficiency, variation in gene effect size and false-positive rate due to cell death from excessive cutting in high copy number regions [6,54,55,56,57,58]. These algorithms include BAGEL [59], CERES [6], CRISPhieRmix [56], CRISPRcleanR [57], HiTSelect [60], JACKS [61], MAGeCK MLE [54], MAGeCK RRA [62] and RSA [63], that showed good performance according to the different experimental settings used [64].

## 5. Gene Essentiality in Estrogen Receptor-Positive Breast Cancers

Two independent studies have been performed to uncover gene essentiality across hundreds of human cancer cell lines, the Achilles project at Broad Institute [6,65] through the DepMap portal [66] and the Sanger Project Score [7,67] whose data can be used to uncover the estrogen signaling related genes. To date, both studies have considered 11 BC cell lines, belonging to the ERα+ subtype (Table 1) and 18,333 and 17,995 genes were independently screened from Broad’s and Sanger’s datasets, respectively.

The reduction of cell viability upon gene inactivation was quantified using individual gene scores across cell lines (gene dependency profiles) using fully processed data available for download from the Meyers et al. [6] and Behan et al. [7] datasets. To measure the effect that arises for each shRNA in the Broad’s datasets, a computational method called CERES, which assesses gene dependency levels from CRISPR-Cas9 screening considering also the copy number specific effect has been developed [6]. For analysis of the Sanger’s datasets, instead, gene-level Bayesian Factors (BFs) were implemented as described in [7]. A gene can be considered essential if the CERES score is ≤ −0.5 for [6] data and shows a score < 0 for [7] data. In total, 2117 and 1095 essential genes can be observed in [6] and [7], respectively, with a high level of concordance between the two datasets, resulting in 960 common essential genes. A summary of the essential genes found in each cell line is available in Table 2.

**Figure 1:**
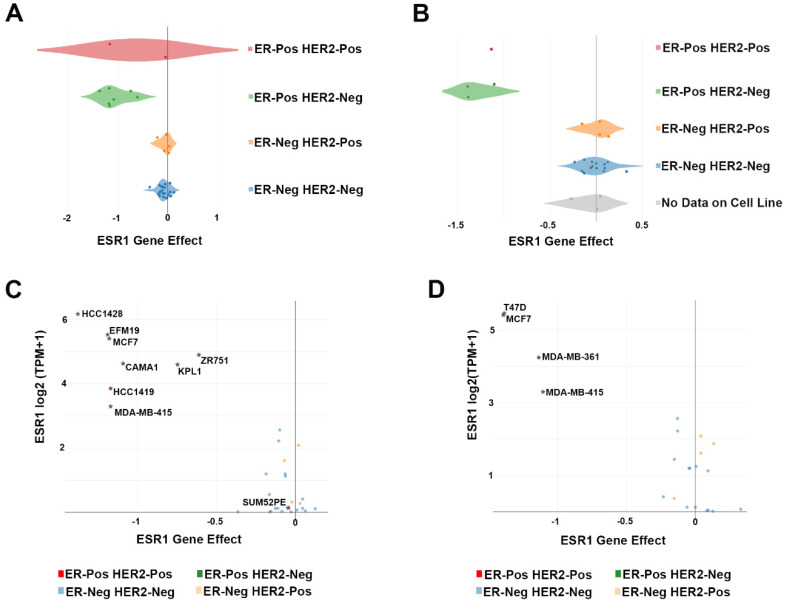
ESR1 essential gene behavior. (**A**,**B**) Box-plots showing the ESR1 essential gene score in all BC cell lines screened in Meyers et al. [6] and Behan et al. [7], respectively. A CERES score value ≤ −0.5 indicates a significant effect of the gene knockout. The gray box-plot represents BC cell lines for which no sub-subtype information is available. (**C**,**D**) Scatter plots showing the correlation between ESR1 gene effect, indicated as the CERES score, and its expression, indicated as log_2_ (TPM+1) for all BC cell lines screened in Meyers et al. [6] and Behan et al. [7], respectively.

The key gene ESR1 shows a significant gene effect score in ERα+ cell lines (except for SUM52PE cells), as displayed in the box-plots of Figure 1A,B where data from Meyer et al. [6] and Behan et al. [7] are considered, respectively. In addition to genome-scale CRISPR-Cas9 screening data, the DepMap portal provides information about drug sensitivity and other several omics data, such as the expression and mutation data of the Cancer Cell Line Encyclopedia (CCLE), together with copy number, methylation, protein array and translocation data [68]. This explores the several relationships with respect to essential genes and to elaborate predictive and comprehensive models for drug sensibility. By comparing ESR1 expression and its dependency score, the gene appears more essential in those cell lines where ESR1 is characterized by a high expression, as shown in Figure 1C,D. In the data related to the GeCKO CRISPR-Cas library, only one cell line, T47D, belongs to ERα+ subtype; it shows 1915 essential genes, with a CERES score ≤ −0.5, of which 724 are shared with those retrieved in common between [6] and [7] datasets. On the other hand, considering the data based on the lentiviral-based pooled RNAi library, 702 essential genes were identified with a DEMETER score ≤ −0.5 among the 13 ERα+ BC cell lines screened. About 66% resulted in common with the 960 genes. The DEMETER score is a computational method developed to analyze RNAi screens. It uses the depletion values induced by each shRNA construct to infer the effect of suppressing its intended target (on-target) and of expressing a given miRNA seed (off-target) in each screened cell line. To assess the problem related to batch effect, variable screen quality and difficulty assessing gene dependency on an absolute scale, DEMETER2 has been developed. In addition to the large-scale CRISPR-Cas9 dropout screenings generated so far, other studies have used CRISPR knockout screenings to investigate genes whose loss affects cell viability. Xiao et al. [12] used GeCKO CRISPR knockout in two ERα+ cell lines, MCF7 and T47D, to assess essentiality also upon hormonal stimulation. They identified 4174 and 3914 essential genes in MCF7 cells under vehicle or estrogen stimulation, respectively, and 4215 and 3430 in T47D following the same experimental approach. Each group of genes shares about 60% of similarity with the set of genes identified in common between Broad and Sanger. In the context of gene essentiality analyses, focused to understand the hormone influence in characteristic phenotype development, and in the further characterization of the mechanism underline endocrine therapy resistance, Nagarajan et al. performed a CRISPR screening in MCF7 [11]. The authors showed how among the known ERα interactors, such as CCND1, GATA3 and FOXA1, ARID1A is a pivotal mediator of the loss of antiestrogen responsiveness. Of the genes resulted essentially 70% resulted in common with the group of 960 essential genes indicated above.

## 6. Functional Pathways Involving Estrogen Receptor-Positive Essential Genes

**Figure 2:**
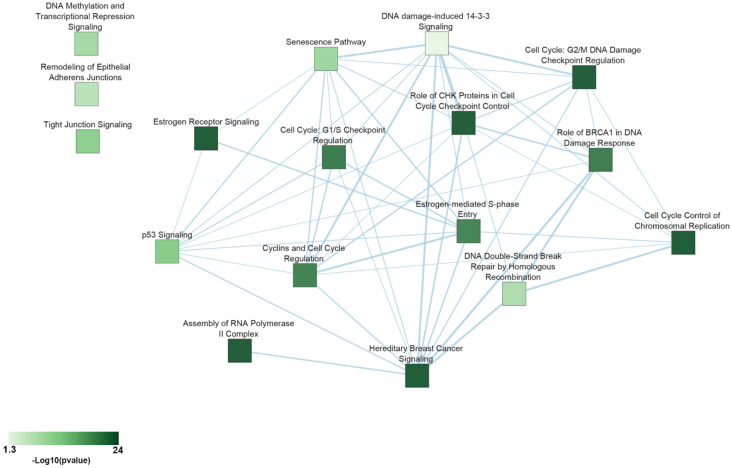
Canonical pathway enrichment analysis involving ERα+ BC essential genes. Network summarizing the canonical pathways involving key genes found essential in the genome-wide dropout screenings considered here. Edges between nodes (light blue lines) were generated using an overlap coefficient of 0.3 and their width is proportional to the number of shared genes.

To explore the pathway landscape where potential drivers for ERα+ BC are involved, functional annotation analysis was performed considering the 960 common essential genes, previously obtained comparing Broad and Sanger screening. The top enriched pathways (B-H *p* < 0.05) show many biological processes relevant in tumorigenesis and cancer progression such as DNA repair mechanisms, cell cycle regulation, epithelial–mesenchymal transitions, DNA methylation, transcriptional repression signaling and senescence pathway as well as, several pathways related to estrogen receptor activity, such as estrogen receptor signaling and estrogen-mediated S-phase entry. These pathways, characterizing the luminal BC phenotype, are listed in Table 3 and displayed as a network, generated using Ingenuity Pathway Analysis and EnrichmentMap, in Figure 2 and involve several essential genes, such as the nuclear respiratory factor-1 (NRF-1), which is a key regulator of mitochondrial gene transcription. It was shown that oxidative stress in hormone-responsive BC cells increases NRF-1 expression and determines a decrease in ERα expression [69]. Moreover, it was observed that NFR-1 phosphorylation is mediated by AKT activation due to the estrogenic increase of ROS levels, contributing to the induction of BC cell growth [70]. Nowadays, more attention is paid to define the role of mitochondria and redox signaling pathways in the cancer cells’ metabolic reprogramming and their apoptotic response to exogenous stressors as therapeutic agents [71]. It has been observed that NRF-1 expression in tamoxifen-resistant BC cells was higher than sensitive BC cells, as well as endocrine-resistant phenotypes associated with a bioenergetics profile much more vulnerable to metabolic stress than endocrine sensitive BC cells. Despite the evidence, the role of NRF-1 in endocrine resistance remains unidentified [72].

Another interesting essential gene involved in the estrogenic pathway is the DEAD box polypeptide 5 (DDX5), an ATP-dependent RNA helicase involved in several fundamental biological processes such as transcription, RNA processing, DNA damage- repair and splicing. This multifaceted activity depends on its capability to act as a coregulator of several oncogenic transcription factors as β-catenin, p53, STAT3 and ERα [73]. Acting as ERα coregulators, DDX5 promote receptor recruitment on estrogen-dependent genes as TFF1 and mediate interaction with (CBP)–p300, steroid receptor coactivator (SRC) family and RNA polymerase II (RNA Pol-II), showing an important role in the transcription machinery assembly [74]. It has also been observed that estrogen receptor phosphorylation by MAPK signaling promotes ERα-DDX5 interaction, which, in turn, enhances AF1 activity. The ratio between coactivators and corepressors by AF-1 domain shows a role in the regulation of BC growth after tamoxifen treatment [75].

Fitness genes identified by CRISPR-Cas screening in ERα+ BC cell lines are also involved in the regulation of DNA methylation associated with transcriptional repression of gene expression. DNA methylation is one of the main mechanisms that translate environment signals in epigenetic modifications leading to reversible changes in the cellular transcriptome. It is now known that aberrations in the DNA methylation are related to BC progression, prognosis, response to treatment and patients outcome [76] making epigenetic changes as one of the hallmarks of BC carcinogenesis. This process is mediated by DNA methyltransferases (DNMTs) enzymes that are methylated of 5′-cytosine residues, mainly contained in CpG sequences (CpG island), largely distributed in the human genome. As another solid tumor, positive BC is associated with global hypomethylation of DNA despite the local hypermethylation of specific genes localized mainly in CpG islands around the transcription start sites. Hypermethylation of promoter CpG islands is often associated with silencing of gene expression because it coincides with a repressive chromatin status where nucleosome occludes the transcription factor binding sites, preventing transcriptional machinery recruitment [77]. In BC, promoter hypermethylation and the consequent silencing were observed in many fundamental genes as BRCA1 involved in DNA repair, CDH1 involved in cell adherence and in the ESR1 gene. Considering that epigenetic inactivation of ERα can be a mechanism of endocrine resistance development, several studies clarified mechanisms that led to ESR1 gene methylation. This latter, in fact, appears methylated in the 5′ region of in ERα-negative BC, contrary to *ESR1* in long-term estradiol deprivation cells, which contain hypomethylated CpG islands and are characterized by increased ERα levels, suggesting a crucial role of methylation in regulation of ERα expression [78].

The association in DNA Methylation and Transcriptional Repression Signaling between fitness gene DNMT1, CHD4 and RBBP4 proteins is noteworthy. DNMT1 is a member of DNA methyltransferase (DNMT) family, responsible to maintain of the methylation status of DNA during cell division while CHD4 (Chromodomain helicase DNA binding protein 4) and RBBP4 (histone chaperone proteins NuRD) belonging to chromatin remodeling NuRD complex. Recently, in colorectal cancer, a strict association between NuRD complex and DNMT proteins was found, suggesting synergic cooperation to regulate epigenetic gene silencing, proposing a combined inhibition of DNMTs and the NuRD complex as a potential novel therapeutic strategy [79]. Considering that specific inhibition by antisense oligonucleotides against DNMT1 leads to restart expression of ERα in negative BC cells, this gene can be an interesting target to evaluate for restoring ERα expression in order to promote endocrine therapy efficiency [80].

DNA damage/repair is a multifactorial biological process composed of different pathways acting simultaneously with the aim to eliminate structural lesions in DNA and maintain genome stability and integrity. However, there is a growing body of literature, which identifies estrogen signaling as regulating key effector DNA damage response (DDR) proteins such as ATM, ATR, p53, BRCA1, and BRCA2, as well as direct interactions with the DNA repair machinery. The presence of DNA damage stimulates the recruiting of different sensor proteins that bind to and signal to cell cycle checkpoint and DNA damage checkpoint kinases as well as the involvement of multiple complexes, complementary and partially overlapping pathways acting to safeguard the genome integrity and the normal cell life cycle [81]. This intricate signaling network is commonly referred to as the DNA damage response (DDR) and it is responsible for monitoring genome health [82]. The importance of the DDR and repair is demonstrated by the fact that some of the genes that are considered essentials for positive BC cell survival encode for components of DNA repair pathways. In this context, RAD51 is responsible to the nucleoprotein filament formation on the single-stranded DNA ends, a crucial step for the homology search and strand invasion during Homologous Recombination (HR) processes, a high-fidelity pathway of BC DNA repair machinery involved into double-strand breaks repair which include also BRCA1 and BRCA2 genes. The role of BRCA1 in HR is based on 5′ to 3′ resection of DSBs to form 3′ ssDNA overhangs and loading RAD51 onto the ssDNA whereas BRCA2 seems to recruit Rad51 onto ssDNA [83]. RAD51 and its related proteins have been found deregulated or overexpressed in different types of BC, such as ERα+ BC, TNBC and Hereditary BC. Its overexpression correlates also with the histologic grade of sporadic and invasive ductal BC when also BRCA1 tumor suppressor function is abolished by the downregulation of the protein levels, underlying that both contribute to the pathogenesis of this type of tumor [84].

## 7. Interaction Proteomics as Tool for Estrogen Signaling Protein Network Dissection

In the field of estrogen-responsive BC being ERα the master regulator of estrogen signaling including a dense network of cointeractors, many proteomic studies have been carried out with the aim of identifying novel ERα-partners involved in the molecular bases of BC progression and to discover novel biomarkers and putative pharmacological targets [11,73,85,86,87,88,89].

With this aim, over the years, several interaction proteomics approaches have been used, for the identification of native protein complexes [90], as well as methodical approaches combining crosslinked molecular complex–chromatin immunoprecipitation with Mass Spectrometry (MS) for the identification of transcriptional cofactors and chromatin-associated proteins, such as Rapid Immunoprecipitation Mass spectrometry of Endogenous protein (RIME) [91]. More recently high-throughput proteomics techniques have been developed that are useful in translational research, such as data-independent acquisition (DIA) [92]. This new approach offers a more sensitive and accurate protein quantification starting from plasma, tissue lysates, blood, and saliva samples. Multiple applications of this technology are based on its association with many purification methods, as for example Surface Adsorption–DIA approach, where intact pathogens are used as a bait to capture the host–pathogen protein interactor [93].

Most of the biological processes modulated by essential gene associated pathways, highlighted in this review, are mainly based on synergic activities of individual proteins. Interacting with each other in a coordinative fashion, these molecules define the functional and biological role of the complex to which they belong. Then, considering the essentiality of ERα in hormone-responsive BC cells as key regulators of biological function required for cellular fitness, the analysis of estrogen receptor network compositions provides useful information about their involvement in molecular mechanisms underlining hormone-responsive BC development and resistance to drug treatments.

The majority of the essential genes identified in common between the studies described in [6] and [7] encode for estrogen receptor interactors

**Figure 3:**
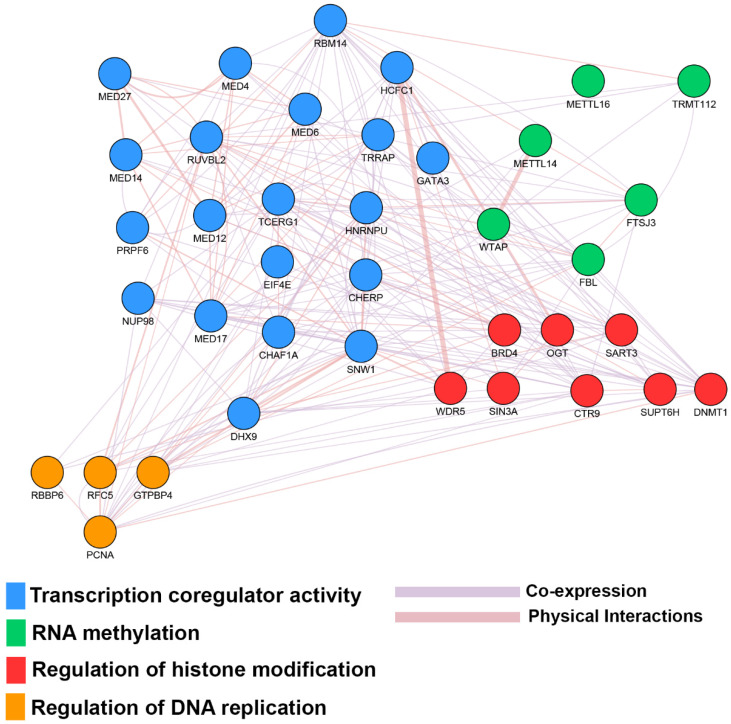
Biological functions involving essential genes in ER+ BC encoding ERα interactors. Network showing the top statistically significant biological functions involving essential genes found in common with ERα interactors. The edges represent physical interactions and coexpression among the proteins. Their width is proportional to the strength of interactions.

To corroborate the key role of these molecules in the context of hormone-responsive BC, a gene ontology analysis was performed. It showed the engagement of ERα molecular partners and in several biological processes essential for the cell growth, differentiation, survivor, tumor development and chemotherapy resistance. Among these functions, those related to the regulation of DNA replication, the epigenetic modulation of RNA methylation, histone modification and transcriptional coregulatory activity, directly related to estrogen signaling are particularly of interest, as shown in Figure 3.

The essential gene RNA helicase A (DHX9) is a protein responsible to regulate transcription in an ATP-dependent manner and involved in chromatin remodeling identified by Tarallo et al. [89]. It regulates transcription of several genes involved in DNA repair, mediating interacting with RNA polymerase II holoenzyme and BRCA1 [94], and it is affected by anti-estrogenic treatment because it is clearly detected in very low quantity after ICI treatment than the E2 condition. The same group enlarged the knowledge of ERα molecular partners performing a purification and MS analysis on nuclear extract pre-treated or not with RNase, further characterizing the receptor interactome [73] FTSJ3 represent another interesting gene. It is a 2′-*O*-Me methyltransferase acting in associations with NIP7 for Ribosome biosynthesis. The protein contains a putative RNA-methyltransferase domain (FtsJ) in the N-terminal region and two uncharacterized domains in the central (DUF3381) and C-terminal (Spb1_C) regions [95]. A recent study demonstrates a functional role of FTSJ3 in promoting BC growth and survival, therefore representing a novel putative target for anticancer treatment [96].

With a RIME-based approach, Mohamed et al. [97] found GREB1 as one of the most essential coregulators for ERα-mediated transcription. Currently, it is demonstrated that GREB1 is necessary for ERα+ tumor growth [98] and its overexpression may increase cell proliferation rate in vitro [99]. The interactor dataset, moreover, comprises other characterized ERα-pioneer factors FOXA1, TLE1, and AP2-g [100], putative pioneer factors such as GATA3 [101], known cofactors that were implicated as ERα-associated protein, and a number of repressors, such as HDAC2, which are known to be recruited with estrogen-ERα to repressed gene such as cyclin G2 [102].

Despite the proven efficacy of ERα targeting in endocrine therapy, resistance phenomena are widely spread. In order to identify novel ER partner proteins, Cirillo et al. [86] used a methodical approach combining TAP purification to MS and identifying different ERα interactomes followed to the treatment with the different classes of ligands in BC cells. The functional analysis of the biological processes involving ERα and the proteins identified, underlined significant differences in biological functions associated with each treatment, reflecting the influence of compounds receptor binding on the recruitment of transcriptional coregulators. In addition, following treatment with tamoxifen, several proteins and the core complex formed by ERα and the pioneer factors FOXA1, GATA3 and TLE1 were found deregulated [97]. Moreover, in the context of endocrine resistance, with the combination of genome-wide CRISPR-Cas9 screening and RIME, it has been possible to discover ARID1A and other SWI/SNF chromatin remodeling complex components as the factors critically involved in drug response against ERα inhibitors, such as tamoxifen and Fulvestrant [11]. There are three known SWI/SNF complexes called BAF, p-BAF and noncanonical BAF (ncBAF) with ARID1A specific subunit for BAF whereas BRG1 and BRM are common in all three [103]. Initial depleted gRNA analysis in different postinfection time points showed widely known essential ERα interactors, including Cyclin D1 (CCND1), FOXA1 and GATA3 [101] even if comparing with different kinetics; subsequent analysis of the gRNA for essential genes involved in tamoxifen and ICI responses show that, despite the two compounds have different mechanisms of action, more than the half genes (63.5%) are common in the MCF7 response to both inhibitors. Among several genes encoding for different BAF components, ARID1A was found most essential for both tamoxifen and ICI activity and its depletion resulted in strong drug resistance to both compounds. Additionally, ARID1A depletion sensitizes cell drug response to JQ1 and other two relevant clinical BET inhibitors, suggesting a critical role of this complex in mediating ERα action and results showed ARID1A and several SWI/SNF components, including BRG1and BRM as physical interactors of the receptor even in the surgical tumor tissue.

Recently, we demonstrated the key role of the previously identified DOT1L (disruptor of telomeric silencing-1-like) ERα partner [86] in hormone-responsive and endocrine-resistant BC cells [87]. DOT1L catalyzes the mono-, di- and trimethylation of Histone H3 on Lysine-79 (K79); it is involved in different biological processes, such as DNA repair, transcriptional elongation and cell cycle progression and its enrollment in cancer progression has been widely demonstrated in different types of cancers, such as in mixed-lineage leukemia (MLL) in which participates in producing MLL fusion proteins, sustaining leukemogenesis processes [104], prostate cancer [105] and ovarian cancer [106]. Our investigation revealed that its silencing or pharmacological inhibition causes a reduced hormone-responsive BC cell proliferation rate and increased cell death both in vitro and in vivo. These phenomena are a direct consequence of a series of inhibition processes on ERα signaling to the genome with a direct inhibitory effect on hormone-dependent genes, including ESR1 itself and some of its cofactors. Interestingly, tamoxifen and ICI endocrine-resistant BC cells showed the same responses to DOT1L blockade both in vitro and in vivo, pointing out DOT1L as a new target in hormone-responsive and endocrine-resistant BC. In addition, with ChIP-MS our group found a series of common interactors between DOT1L and ERα, resulting in fitness genes in BC cells analyzed here [70]. Among these, BRD4, a member of the BET protein family able to bind nonhistone-proteins in acetylated chromatin influencing the access of the transcriptional regulators; it actively participates in transcriptional elongation interacting via CDK9/pTEFb [107]. Recently, targeting BRD4 has been demonstrated effective in reducing tumor migration and invasion in vitro and in the PDX model and for the lung colonization of BC cells [108]. Moreover, BRD4 is a well-defined target of BET family inhibitors JQ1, representing valid therapeutic alternatives in tamoxifen-resistant BC [109].

## 8. Conclusions

BC, and in particular its ERα+ subtype is a heterogeneous pathology showing specific molecular features, where the endocrine resistance represents the major challenge for the management of patients affected by this disease. This cancer diversity depends on several genomic features involved in BC development and resistance; cancer progression is encoded by a variety of genes involved in several pathways, some belonging to several networks able to evolve in resistance mechanisms. In this context, the genome-wide CRISPR/Cas9 approach can help in deciphering and overcoming these mechanisms pointing out essential genes involved in functional pathways for ERα+ BC biology. These include relevant signaling features, such as cell cycle regulation, DNA repair, methylation and the estrogen receptor signaling (Figure 2), which is one of the most affected, confirming the essential role of ERα and its molecular partners in BC progression. Concerning the ability of this receptor subtype to assemble in functional multiprotein complexes, many ERα interacting partners are also essential genes involved in biological processes relevant to BC progression (Figure 3). In conclusion, the estrogen signaling blockade by targeting one or more ERα+ BC essential genes, provide new insight to better understand the molecular basis of this pathology, and these BC essential genes can be used as new possible targets exploitable in endocrine-therapy-resistant BC management.

## Figures and Tables

**Table 1:** List of the 11 BC ERα+ cell lines screened in Meyers et al. [6] and/or Behan et al. [7].

Cell Line	Lineage	Lineage Subtype	Lineage Sub-Subtype	Tumor Type	CRISPR Screening
CAMA1	Breast	Breast Carcinoma	ER-Pos HER2-Neg	Metastasis	[6]
EFM19	Breast	Breast Ductal Carcinoma	ER-Pos HER2-Neg	Primary	[6]
HCC1419	Breast	Breast Ductal Carcinoma	ER-Pos HER2-Pos	Metastasis	[6]
HCC1428	Breast	Breast Carcinoma	ER-Pos HER2-Neg	Metastasis	[6]
KPL1	Breast	Breast Carcinoma	ER-Pos HER2-Neg	Metastasis	[6]
MCF7	Breast	Breast Carcinoma	ER-Pos HER2-Neg	Metastasis	[6,7]
MDA-MB-361	Breast	Breast Carcinoma	ER-Pos HER2-Pos	Metastasis	[7]
MDA-MB-415	Breast	Breast Carcinoma	ER-Pos HER2-Neg	Metastasis	[6,7]
SUM52PE	Breast	Breast Carcinoma	ER-Pos HER2-Pos	Metastasis	[6]
T47D	Breast	Breast Ductal Carcinoma	ER-Pos HER2-Neg	Metastasis	[7]
ZR751	Breast	Breast Ductal Carcinoma	ER-Pos HER2-Neg	Metastasis	[6]

**Table 2:** Number of essential genes in ERα+ breast cancer cell lines.

Project	ERα+ BC Cell Line	Number of Essential Genes
[6]	CAMA1	2292
	EFM19	2278
	HCC1419	2279
	HCC1428	2042
	MCF7	2463
	MDA-MB-415	2162
	KPL1	2305
	SUM52PE	3089
	ZR75.1	2149
[7]	MDA-MB-361	1494
	MDA-MB-415	1156
	MCF7	761
	T47D	1191
[9]	T47D	1915
[10]	BT474	433
	EFM19	515
	HCC1428	804
	HCC1500	817
	KPL1	794
	MCF7	527
	MDA-MB-175VII	771
	MDA-MB-361	697
	MDA-MB-415	415
	T47D	803
	UACC812	744
	ZR75.1	799
	ZR75.30	510

**Table 3:** Canonical pathway analysis performed on ER+ BC essential genes.

Pathway	*p*-Value	Essential Genes
Cell Cycle Control of Chromosomal Replication	5.01 × 10^−24^	*CDC45*, *CDC6*, *CDC7*, *CDK1*, *CDK11A*, *CDK4*, *CDK7*, *CDK9*, *CDT1*, *DBF4*, *MCM2*, *MCM3*, *MCM4*, *MCM5*, *MCM6*, *MCM7*, *ORC1*, *ORC6*, *PCNA*, *POLA1*, *POLA2*, *POLD1*, *POLE*, *PRIM1*, *RPA1*, *RPA2*, *RPA3*, *TOP2A*
Assembly of RNA Polymerase II Complex	3.16 × 10^−15^	*CCNH*, *CDK7*, *DR1*, *ERCC3*, *GTF2A1*, *GTF2A2*, *GTF2B*, *GTF2E1*, *GTF2E2*, *POLR2B*, *POLR2C*, *POLR2D*, *POLR2E*, *POLR2F*, *POLR2G*, *POLR2H*, *POLR2I*, *POLR2K*, *POLR2L*, *TAF1*
Hereditary Breast Cancer Signaling	3.16 × 10^−11^	*ATR*, *CCND*, *CDK1*, *CDK4*, *CHEK1*, *KRAS*, *PIK3CA*, *POLR2B*, *POLR2C*, *POLR2D*, *POLR2E*, *POLR2F*, *POLR2G*, *POLR2H*, *POLR2I*, *POLR2K*, *POLR2L*, *RAD51*, *RFC3*, *RFC5*, *RPA1*, *RPS27A*, *SMARCB1*, *SMARCE1*, *TUBG1*, *UBA52*, *WEE1*
Estrogen Receptor Signaling	1.32 × 10^−6^	*CCND1*, *DDX5*, *EIF2B1*, *EIF2B2*, *EIF2B3*, *EIF2B4*, *EIF2B5*, *EIF4E*, *ESR1*, *FOXA1*, *KRAS*, *MED12*, *MED14*, *MED17*, *MED18*, *MED20*, *MED21*, *MED27*, *MED30*, *MED31*, *MED4*, *MED6*, *MTOR*, *MYC*, *NRF1*, *PCNA*, *PIK3CA*, *POLR2B*, *PPP1CB*, *PPP1R12A*, *SDHC*, *TFAM*, *TRRAP*, *UQCRFS1*
Role of CHK Proteins in Cell Cycle Checkpoint Control	2.13 × 10^−5^	*ATR*, *CDK1*, *CHEK1*, *CLSPN*, *PCNA*, *PLK1*, *PPP2CA*, *RAD17*, *RFC3*, *RFC5*, *RPA1*
Cell Cycle: G2/M DNA Damage Checkpoint Regulation	3.01 × 10^−5^	*ATR*, *AURKA*, *CDK1*, *CDK7*, *CHEK1*, *PKMYT1*, *PLK1*, *SKP1*, *TOP2A*, *WEE1*
Cell Cycle: G1/S Checkpoint Regulation	4 × 10^−4^	*ATR*, *CCND1*, *CDK4*, *GNL3*, *MYC*, *PAK1IP1*, *RPL11*, *RPL5*, *SIN3A*, *SKP1*
Role of BRCA1 in DNA Damage Response	5 × 10^−4^	*ATR*, *CHEK1*, *PLK1*, *RAD51*, *RBBP8*, *RFC3*, *RFC5*, *RPA1*, *SMARCB1*, *SMARCE1*, *TOPBP1*
Cyclins and Cell Cycle Regulation	6 × 10^−4^	*ATR*, *CCNA2*, *CCND1*, *CCNH*, *CDK1*, *CDK4*, *CDK7*, *PPP2CA*, *SIN3A*, *SKP1*, *WEE1*
Estrogen-mediated S-phase Entry	6 × 10^−4^	*CCNA2*, *CCND1*, *CDK1*, *CDK4*, *ESR1*, *MYC*
p53 Signaling	2 × 10^−3^	*ATR*, *BCL2L1*, *BIRC5*, *CCND1*, *CCNK*, *CDK4*, *CHEK1*, *GNL3*, *PCNA*, *PIK3CA*, *TOPBP1*
Tight Junction Signaling	5 × 10^−3^	*CDC42*, *CDK4*, *CPSF2*, *CPSF3*, *CPSF6*, *CSTF3*, *GOSR2*, *NAPA*, *NSF*, *NUDT21*, *PPP2CA*, *RAC1*, *STX4*, *SYMPK*, *YKT6*
Senescence Pathway	0.01	*ANAPC1*, *ANAPC10*, *ANAPC11*, *ANAPC2*, *ANAPC4*, *ANAPC5*, *ATR*, *CCND1*, *CDC16*, *CDC23*, *CDC26*, *CDC27*, *CDK1*, *CDK4*, *CHEK1*, *EIF4E*, *KRAS*, *MTOR*, *PIK3CA*, *PPP2CA*
DNA Methylation and Transcriptional Repression Signaling	0.01	*CHD4*, *DNMT1*, *RBBP4*, *SAP18*, *SIN3A*
DNA Double-Strand Break Repair by Homologous Recombination	0.02	*POLA1*, *RAD51*, *RPA1*
Remodeling of Epithelial Adherens Junctions	0.02	*ACTR2*, *DNM1L*, *DNM2*, *TUBA1B*, *TUBA1C*, *TUBB*, *TUBG1*
DNA damage-induced 14-3-3 Signaling	0.04	*ATR*, *CDK1*, *RAD17*

## Abbreviations

AIs	Aromatase Inhibitors
BC	Breast Cancer
BFs	Bayesian Factors
CRISPR	Clustered Regularly Interspaced Short Palindromic Repeats
DIA	Data-Independent Acquisition
DSB	Double-Strand Break
ERα+ BC	Estrogen Receptor alpha positive breast cancer
ERα− BC	Estrogen Receptor alpha negative breast cancer
ERE	Estrogen Response Element
FDR	False Discovery Rate
ICI	Fulvestrant, ICI 182780
HR	Homologous Recombination
RIME	Rapid Immunoprecipitation Mass spectrometry of Endogenous protein
RNAi	RNA interference
SERDs	Selective Estrogen Receptor Downregulators
SERMs	Selective Estrogen Receptor Modulators
sgRNA	Single guide RNA
TAP	Tandem Affinity Purification
tracrRNA	Trans-activating crispr RNA
